# High-Resolution FBG-Based Fiber-Optic Sensor with Temperature Compensation for PD Monitoring

**DOI:** 10.3390/s19235285

**Published:** 2019-11-30

**Authors:** Mohsen Ghorat, Gevork B. Gharehpetian, Hamid Latifi, Maryam A. Hejazi, Mehdi Bagheri

**Affiliations:** 1 Department of Electrical Engineering, Amirkabir University of Technology, Tehran 158754413, Iran; mohghoco@gmail.com (M.G.); grptian@aut.ac.ir (G.B.G.); 2 Laser and Plasma Research Institute and Physics Department, Shahid Beheshti University, Tehran 1983969411, Iran; latifi@sbu.ac.ir; 3 Department of Electrical and Computer Engineering, University of Kashan, Kashan 8731753153, Iran; mhejazi@kashanu.ac.ir; 4 Department of Electrical and Computer Engineering, Nazarbayev University, Nur-Sultan 010000, Kazakhstan; 5 National Laboratory Astana, Center for Energy and Advanced Material Science, Nazarbayev University, Nur- Sultan 010000, Kazakhstan

**Keywords:** partial discharge, fiber bragg grating, fiber optic sensor, acoustic wave

## Abstract

This paper presented a new sensor to detect and localize partial discharge (PD) in power transformers based on a fiber Bragg grating (FBG). The fundamental characteristics of the proposed sensor, as a PD detector, were temperature compensation and direction independence. The proposed high-resolution PD detector operated based on the FBG wavelength shift. It is necessary to evaluate the physical parameters of the sensor to achieve the best results. Therefore, in this paper, the detected signal strength was investigated for different angles and temperatures. A Teflon hollow mandrel and two FBGs attached to the inner and outer surfaces of the hollow mandrel were chosen as the inner transformer PD detector. The changes in the sensor output were less than 0.4 mV and 0.5 mV for direction variations and a temperature variation of 14 °C (degrees Celsius), respectively. Consequently, the proposed sensor could be successfully employed for the detection of a transformer PD signal.

## 1. Introduction

Partial discharge (PD) is a destructive phenomenon in power transformers. Researchers have investigated different measurement techniques to evaluate the physical and chemical effects of PD.

Detecting the PD acoustic wave inside and outside a transformer is a well-known technique. The detection and localization of PD by an acoustic wave should have high precision. Fiber-optic sensors are a good candidate for internal sensors because they are sensitive to acoustic signals and insensitive to other electromagnetic waves as they do not conduct electrons [1,2,3]. This technique can be performed online and is nondestructive. An important consideration for internal sensors is their temperature dependence. The temperature of oil exhibits different behavior in various locations of the transformer tank, depending on the loading. Therefore, regardless of their position in the transformer, the sensors should achieve the same performance.

Mach–Zehnder, Fabry–Perot, Sagnac, and Michelson interferometers are different types of fiber-optic interferometers that are used for sensing. The Mach–Zehnder interferometer is a popular interferometer that is employed to detect PD signals [4,5,6]. A fiber coil is the basis of most sensors in such interferometric techniques. Due to the long fiber length in the coil, a high resolution can be obtained [7], but the noise level increases. It is worth noting that temperature is also a form of noise that has negative effects on the sensing results.

Many researchers have studied the diaphragm fiber of Fabry–Perot interferometry to detect PD signals [8,9,10,11,12,13]. The material of the diaphragm and the dimensions of the cavity and diaphragm are parameters that must be optimized. The effectiveness of the diaphragm sensor completely depends on the signal direction. This direction dependence causes the output of the sensor to vary for different angles, thus limiting the detection direction. As a result, it is necessary to increase the number of sensors to detect the PD at all locations in the transformer. Another drawback is the narrow bandwidth of the diaphragm sensor and can cover part of a wide range of PD acoustic signal frequencies.

In recent years, there has been considerable interest in the use of fiber bragg grating (FBG) as a PD sensor [14,15,16,17,18,19]. Fiber bragg grating (FBG) sensors are reliable and stable, exhibit nearly flat and linear responses to sensing parameters, and have been used to sense different parameters, such as temperature, strain, pressure. FBG could be designed in specific applications by developing the technology. FBG is attached to Mandrel and cantilever with special glue to sense mechanical waves [20,21]. Michelson and Sagnac interferometers [22,23,24] and different types of fiber, such as multimode fiber and a long-period grating (LPG) [16,17,25,26], have also been used to detect PD signals.

Acoustic pressure decreases with distance. Therefore, it is necessary to amplify acoustic waves before detection. A mandrel and a cantilever are two mechanical amplifiers that are used to amplify acoustic waves [10,27,28]. The frequency bandwidth of the cantilever is narrow, but there are different resonating modes in the mandrel, covering a wide bandwidth. Optical and electrical amplifiers, such as the erbium-doped fiber amplifier (EDFA) and an operational amplifier (OPAMP), have been used to amplify such signals [29,30].

This paper presented a new solution for transformer monitoring with a high-resolution fiber-optic sensor that is independent of temperature and the receiving signal direction.

The sensor combines two FBGs and a Teflon mandrel, which significantly improves the sensitivity of the sensor. The proposed sensor consisted of two FBGs attached to the inner and outer surfaces of the mandrel. The two FBGs were used in both transmission and reflection modes, and based on the proposed structure, the temperature was compensated, and the sensitivity was doubled. The mandrel was used as a mechanical amplifier to amplify the received acoustic signal based on its resonance frequency and transmitted the amplified received an acoustic signal to FBGs. The sensitivity of the sensor was calculated, and the resonance frequency and angle and temperature variation responses were measured. It was shown that an acoustic PD signal could be successfully detected by the proposed sensor.

## 2. Theoretical Background

### 2.1. The Mandrel and Mechanical Vibration

A mandrel is often used as a PD acoustic amplifier because it covers the broad frequency bandwidth that is required for detection. A mandrel is a hollow cylindrical object that can be fabricated with different materials. The physical properties of the material, such as Young’s modulus, Poisson’s ratio, the density, and the dimensions of the pipe, affect the resonance frequency. The radial resonance frequencies of a shell with n and m modes are given by [10,31]:

(1) $$\omega_{m،n}^{2}=\frac{E}{\rho \left(1-\nu^{2}\right)R^{2}}\left(\frac{\left(1-\nu^{2}\right)\lambda^{4}+a^{2}{\left(\lambda^{2}+n^{2}\right)}^{4}}{n^{2}+{\left(\lambda^{2}+n^{2}\right)}^{2}}\right)\;\;\;$$

where m is the number of axial half-periods, n is the node of the circumferential waves, ρ is the density, ϑ is Poisson’s ratio, L is the length of the mandrel, $\lambda =\frac{m\pi R}{L}$, and $a^{2}=\frac{t^{2}}{12R^{2}}$.

The mandrel considered in this study is made of Teflon. One has the option of choosing the parameters of the mandrel, such as its diameter. In this paper, Young’s modulus was selected considering the material of the mandrel. The diameters and thickness were selected, considering the slicing limitations of the workshop used for fabrication. Using Young’s modulus, the diameters, and the thickness, the length of the mandrel was calculated. In the proposed sensor, the length of the mandrel was 30 mm, with an inner diameter of 11 mm and an outer diameter of 12 mm [10]. The resonance frequencies were calculated according to (1) for m = 1 to 10 and n = 0 to 10, and there were 110 resonance frequencies between 23 kHz and 364 kHz. The resonance frequency decreased when the mandrel was immersed in a liquid because of its added mass. The range of the PD acoustic frequencies was 10 kHz to 300 kHz. Therefore, it was concluded that the almost entire frequency range was covered by this mandrel.

Simulations were performed to calculate the resonance frequency of the mandrel while it was immersed in air and water. Pressure acoustics were used as a physics of study in this software. The dimensions of the acoustic transducer (200LM450, prowave company, New Taipei, Taiwan) relative to sensor size were big. Therefore, the plate acoustic source with a piezoelectric base was used. The sinusoidal acoustic pressure source was located 10 cm from the air-backed mandrel and had an amplitude of 100 Pa at different frequencies. Density, Young’s modulus, and Poisson’s ratio of Teflon were considered as 2200 $\frac{\mathrm{kg}}{m^{3}}$, 0.5 GPa, and 0.46, respectively.

**Figure 1:**
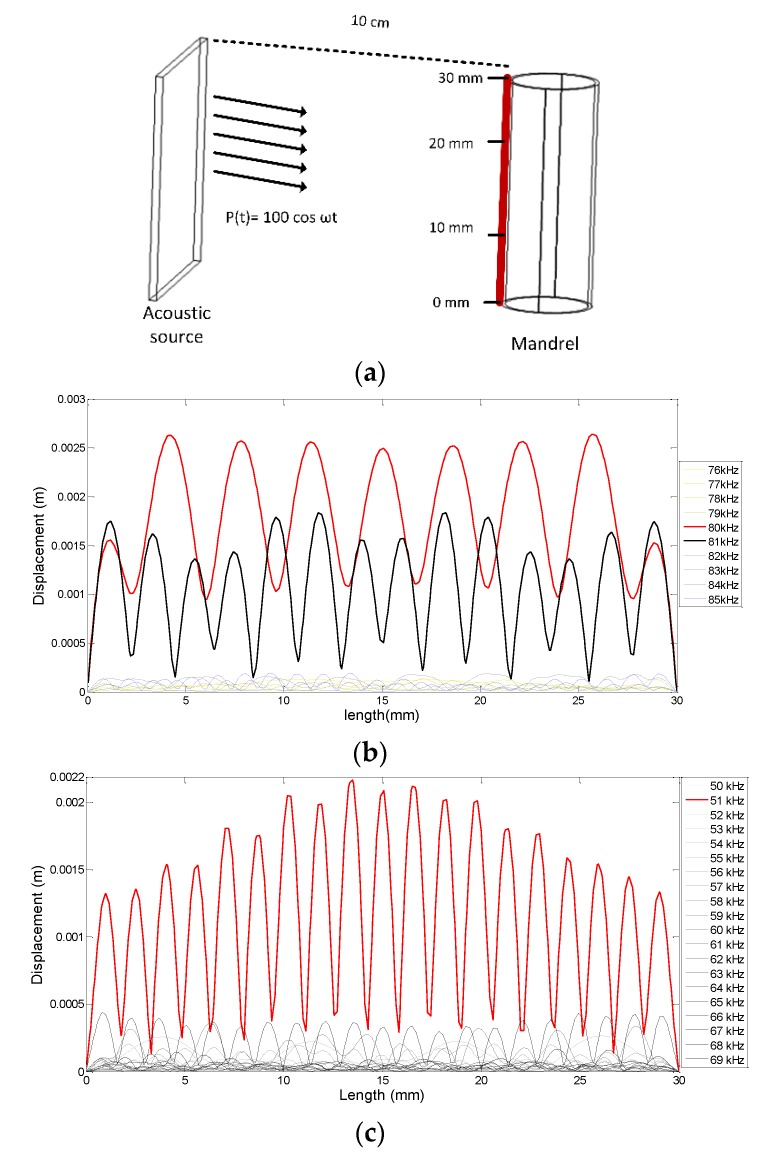
(**a**) Mandrel simulation and (**b**) mandrel shell displacement in water and (**c**) in air.

The mandrel shell displacement was measured on the red line on the mandrel shell, as illustrated in Figure 1a. The simulation results in Figure 1b,c show the mandrel shell displacement on the red line for different frequencies. They showed that the mandrel resonance frequency was approximately 80 kHz and 51 kHz in air and water, respectively. As predicted, the simulation results were in good agreement with the theoretical results.

### 2.2. Principle of the FBG

An FBG is a fiber that includes different reflective sections. The Bragg wavelength (λ) is determined based on the grating period (Λ) and the effective refractive index (neff) [15] by (2). In this paper, two similar FBGs were used in reflection and transmission modes.

(2) $$\lambda_{B}=2n_{eff}\Lambda \;$$

## 3. Experimental Setup

**Figure 2:**
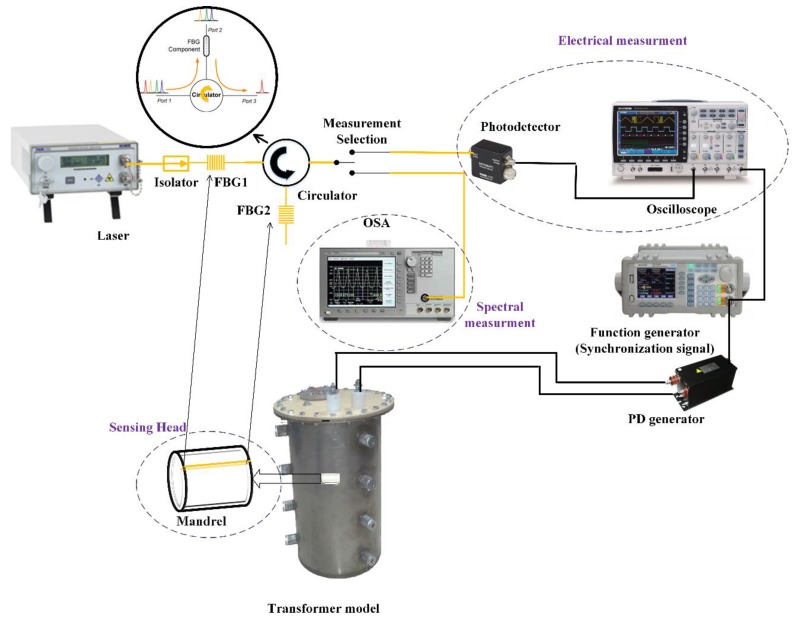
Experimental setup.

A schematic of the test setup is illustrated in Figure 2. A broadband laser (S1FC1550, Thorlabs company, New Jersey, NJ, USA) was used as the laser source, and with a circulator, a feedback loop was designed for the photodetector to receive an optical signal. The photodetector transformed an optical signal into an electrical signal, and the data were recorded by an oscilloscope. The sensor consisted of two FBGs, which were attached to the outer and inner surfaces of the hollow mandrel shell. The broadband laser passed through FBG1, and the transmitted spectrum of FBG1 passed through FBG2. The reflected spectrum of FBG2 was selected by the circulator. For proper sensor operation, the two FBGs must be selected such that their optical spectra match. The concave point of the transmitted spectrum and the peak point of the reflected spectrum occurred at the same wavelength when the sensor was in the initial state.

The acoustic signal pressure shifted the Bragg wavelength of the FBG. Due to the location of the FBG on the inner and outer of the mandrel shell, the wavelength shifts for FBG1 and FBG2 occurred in opposite directions. When the mandrel shell was concave, the outer FBG was stretched, the inner FBG was compressed, and vice versa, when the mandrel shell was convex. Therefore, sensor sensitivity was doubled. The photodetector measured the area under the overlap of the spectra of the two FBGs. Because the spectra of the two FBGs shifted in opposite directions, when the overlap of the spectra increased or decreased, the output voltage increased or decreased, respectively.

**Figure 3:**
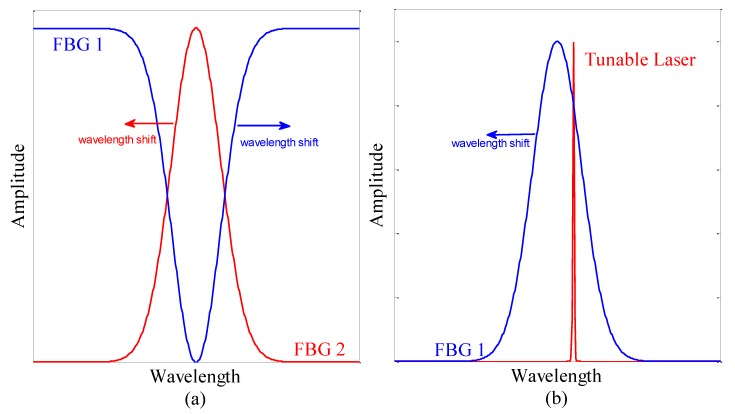
Sensor performance when using (**a**) two FBGs and (**b**) one FBG. FBG, fiber Bragg grating.

Another setup was implemented by one FBG and a tunable laser [21]. The tunable laser generates a narrow spectral bandwidth whose wavelengths can be tuned according to the FBG. However, in this setup, the wavelength of the tunable laser was fixed, and the wavelength of the FBG shifted in response to changes in the sensing parameter. With this setup, the sensitivity was reduced, and some other advantages were lost. Figure 3 shows the FBG spectra for the setups with one FBG and two FBGs.

Teflon rods were installed inside the transformer model and were inserted into the mandrel to keep them fixed. Considering the sensor materials, which are glass (Dielectric strength of FBG ≈2000–3000 kV/inch) and Teflon (Dielectric strength of mandrel ≈1500 kV/inch) in comparison with transformer oil (Dielectric strength ≈400 kV/inch), it was obvious that there was no insulation coordination problem for this sensor.

To model the transformer and create a PD signal in practice, cylindrical steel was made with a diameter of 30 cm and a height of 40 cm. The winding and core were also embedded in the model. The PD was created by a needle-needle electrode fed by a 12/0.22 kV voltage transformer. The distance between these needles could be adjusted regarding the output voltage. In these tests, a calibrated source should be used; thus, based on the frequency range of the PD acoustic wave (10 kHz–300 kHz), an acoustic transducer (200LM450) was used. The sensor was placed at the center of the transformer model, and the distance between the sensor and acoustic source varied according to the purpose of the test.

A synchronization signal should be used to generate and detect PD signals. The synchronization signal was created by a function generator and used to trigger the PD generator and oscilloscope. The PD acoustic wave propagated through the oil, and the sensor detected the acoustic signal. The output of the photodetector was recorded by a 2GS/s GDS-2304 oscilloscope over a 100 ms period.

Considering the importance of PD detection for utility engineers, it was assumed that the sensor was installed during the transformer production phase to ensure that online PD detection was possible. Additionally, it was possible to use this sensor for other transformers that were already installed in substations.

## 4. Sensitivity

In this section, the theoretical sensitivity of the sensor to the input pressure was analyzed. The mandrel height, thickness, and inner radius were L, T, and r, respectively.

**Figure 4:**
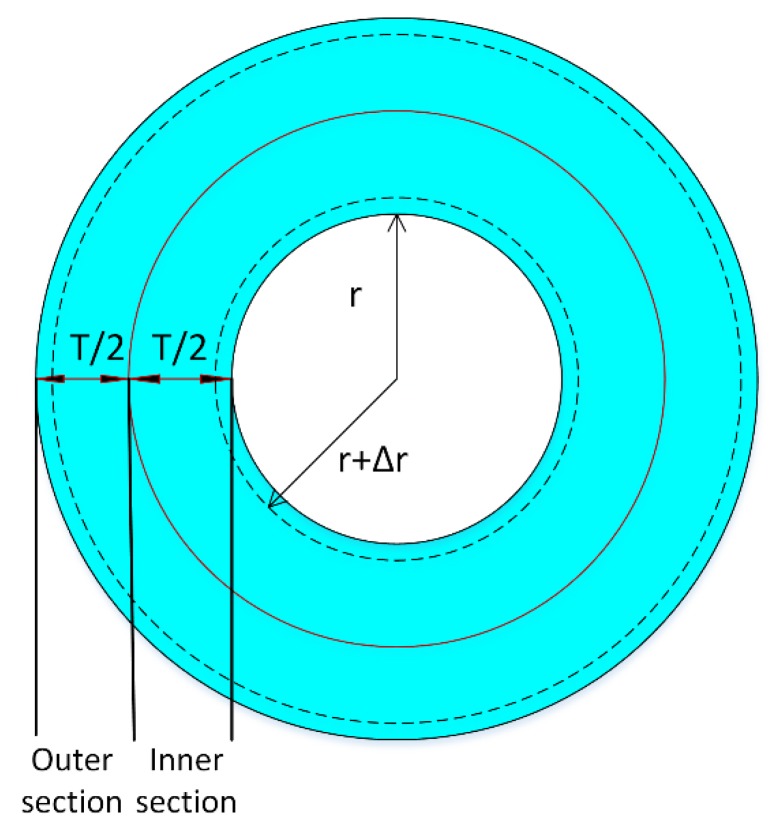
Mandrel cross-sectional view.

To simplify the sensitivity calculation, the mandrel shell was divided into inner and outer sections with equal thicknesses of $\frac{T}{2}$ (Figure 4). It was assumed that the acoustic pressure surrounding the mandrel was $p\left(t\right)=p_{A}\cos\left(wt\right)$ and that the volume changes in the inner section due to pressure were calculated as follows:

(3) $$\Delta V=\frac{PV}{B}=P\frac{\pi [\left(r+\frac{T}{2}{)}^{2}-r^{2}\right]l}{B}\;$$

V and ΔV were the initial volume and volume change in the inner section, and B was the bulk modulus:

(4) $$\pi [\left(r+\frac{T}{2}{)}^{2}-{\left(r+\Delta r\right)}^{2}\right]l=\pi [\left(r+\frac{T}{2}{)}^{2}-r^{2}\right]l-\Delta V$$

Using Equations (3) and (4), we obtained:

(5) $$2r\Delta r+\Delta r^{2}=\frac{P}{B}\left(rT+{\frac{T}{4}}^{2}\right)$$

Higher-order terms of the radius change were neglected, therefore:

(6) $$\Delta r=\frac{P}{B}\frac{\left(rT+\frac{T^{2}}{4}\right)}{2r}$$

The strain in the axial direction for the inner FBG was calculated as follows:

(7) $$\varepsilon_{z_{2}}=\frac{\Delta L_{1}}{L_{1}}=\frac{\Delta r}{r}=\frac{P}{B}\frac{\left(rt+\frac{t^{2}}{4}\right)}{2r^{2}}$$

where L_1_ was the initial length of the inner FBG, and the wavelength shift due to the strain in the axial direction was calculated by [32]:

(8) $$\frac{\Delta \lambda_{Bragg1}}{\lambda_{Bragg1}}=\left(1-p_{ei}\right)\varepsilon_{1}$$

where ∆λBragg1 and λBragg1 were the Bragg wavelength shift and wavelength, respectively, for FBG1. *pei* was the elasto-optical coefficient of silica fiber and was equal to 0.22 [31];

(9) $$\Delta \lambda_{Bragg2}=0.78\lambda_{Bragg2}\frac{P}{B}\frac{\left(rT+\frac{T^{2}}{4}\right)}{2r^{2}}$$

Same calculation could be used to demonstrate that:

(10) $$\Delta \lambda_{Bragg1}=0.78\lambda_{Bragg1}\frac{p}{B}\frac{\left(rT+\frac{3}{4}T^{2}\right)}{2\left(r+T\right)}$$

Sensitivity is given in Equation (11):

(11) $$Sensitivity=\frac{\Delta \lambda_{Bragg1}+\Delta \lambda_{Bragg2}}{p}\;\;\;$$

## 5. Pressure Test

**Figure 5:**
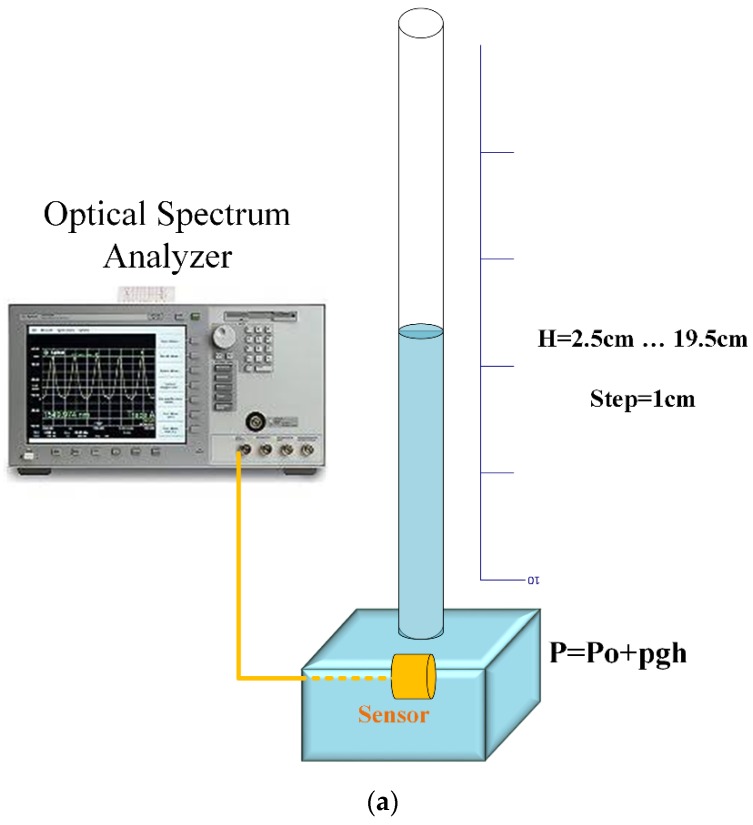

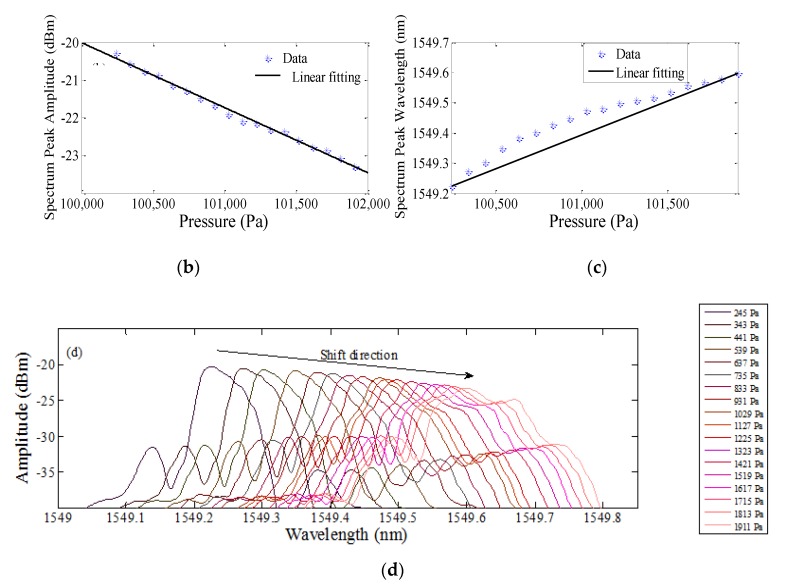
Sensor output due to static pressure. (**a**) Schematic setup for static pressure. (**b**) Change in the spectrum amplitude due to pressure. (**c**) Change in the spectrum wavelength due to pressure. (**d**) Change in the spectrum due to pressure.

First, the sensor performance, which consists of two FBGs and a mandrel, was examined under static pressure. In real applications, the sensor is located in the transformer, and, considering its position, it must tolerate different static pressures of transformer oil. Therefore, feature identification under static pressure must be conducted in advance. The sensor was located in a container in accordance with Figure 5a. The setup was similar to that of a manometer. The pressure was adjusted by setting the water height (h); thus, by changing pressure, the wavelength shifted. The spectra were measured using an optical spectrum analyzer (OSA). It is interesting to note that by increasing the pressure, the amplitude of the spectrum decreased simultaneously with the wavelength redshift, as shown in Figure 5. Notably, Figure 5a,b reveals linear relations between the wavelength variation and pressure and between the spectrum amplitude and pressure, and their slopes were 0.037 nm/kPa and 0.306 db/kPa, respectively. The value of the limit of detection (LOD) under static pressure was equal to 27 Pa.

**Figure 6:**
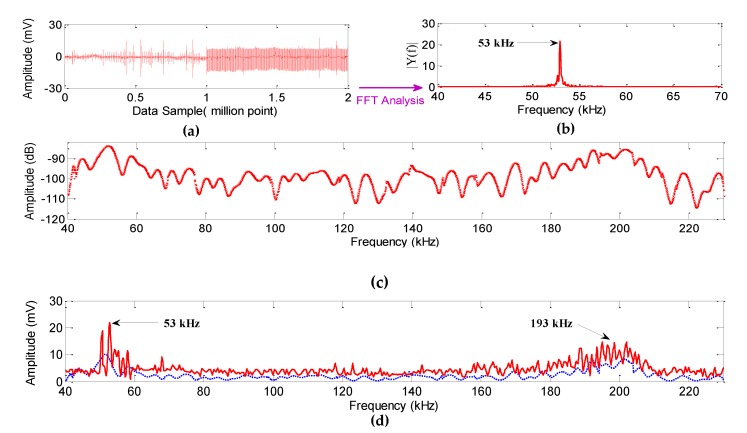
Sensor output. (**a**) Data series detected the signal at 53 kHz. (**b**) FFT analysis of the data series at 53 kHz. (**c**) Sensor output at each frequency step. (**d**) Interpolation of the sensor output for one FBG (blue curve) and two FBGs (red curve).

To evaluate the dynamic pressure performance, the sensor was analyzed in the frequency domain. An acoustic frequency range of 40 kHz to 230 kHz was generated in steps of 0.5 kHz at a distance of 10 cm from the sensor by the piezoelectric transducer (200LM450). The output voltage of the photodetector was recorded at each step by an oscilloscope. The detected time series data and its FFT for a 53 kHz acoustic source are shown in Figure 6a,b. Figure 6c illustrates the amplitude of the FFT spectrum of the detected signals at each frequency step. These tests were repeated 10 times, and after interpolating the data points, the frequency responses are shown in Figure 6c.

Two curves are shown in Figure 6d. The red curve corresponded to the mandrel with inner and outer attached FBGs, and the dashed blue curve corresponded to the mandrel with only an outer attached FBG and the tunable laser as the light source. There were local maxima in these curves. The mandrel resonance frequencies were the main cause of these local maxima. The fluctuation in the red curve was significantly larger than that in the blue curve due to the greater sensitivity of this sensor, as expected, and proved in Section 2.

According to Figure 6, there were two main local maxima at 53 kHz and 193 kHz in each curve. The maximum mandrel shell displacements occurred at 53 kHz, so this frequency was more significant. The experimental and simulation results were consistent with each other. Another main maximum occurred at 193 kHz because the acoustic pressure was the highest at this frequency due to the piezoelectric resonance. According to the acoustic frequency range of PD and signals detected in Figure 6, the sensor performance was considerably acceptable. A major portion of the PD signal could be detected below 230 kHz; hence, the sensor could cover the desired range of the frequency using the mandrel. The FBG was placed in the middle of the mandrel, and based on the shell deformation and displacement (which may be concave or convex), the sensor output varied even for acoustic close frequencies. The low-level output of the sensor at certain frequencies was due to the low pressure of the source. Therefore, the proposed sensor had a broadband frequency response.

The value of the background noise was 0.08 mV, and the acoustic pressure of the piezoelectric transducer was 149 dB (0 dB for 1 uPa/1 V at 100 cm) at 222.5 kHz. The minimum received sensitivity was –113 dB; therefore, the sensor could detect at least 35 Pa at 225.5 kHz. The best resolution for the sensor, which consists of one FBG [21], was 75 Pa in this method.

According to Figure 6, the proposed sensor could cover the PD frequencies, which is a significant advantage over the sensors introduced so far with narrow bandwidths. By using two FBGs, sensitivity and resolution were increased. The resolution for static and dynamic pressures enabled us to identify PD in the initial stage.

## 6. Temperature Dependence

**Figure 7:**
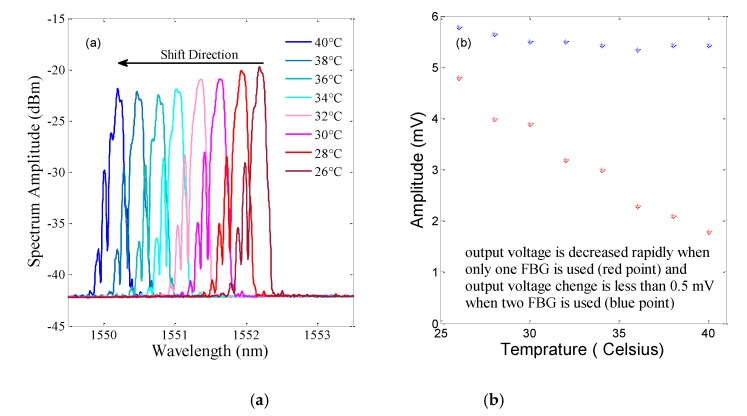
Output change due to a variation in temperature. (**a**) Wavelength shift for the proposed sensor. (**b**) Voltage change with the one-FBG configuration (red point) and the proposed sensor (blue point).

The sensor robustness to temperature is up to 120 °C, considering its materials, so the transformer oil temperature cannot disrupt the sensor function. The aim of the proposed sensor was PD detection with temperature compensation. The sensor wavelength shift was monitored by changing the temperature. The sensor was located in a water container and placed under static pressure of approximately 2 Pa plus atmosphere pressure. In addition, the water temperature was increased from 26 to 40 degrees in steps of 2 degrees. The wavelength shift and voltage change of the proposed sensor due to the increasing temperature are depicted in Figure 7. The overlap of the spectra shifted, but the area under the curve did not change because the two spectra shifted simultaneously. Therefore, there was no change in the sensor output voltage. Figure 7b shows a clear trend in the output, where the change in the output voltage was less than 0.5 mV for temperature variations of 14 degrees Celsius. The output voltage exhibited slight changes due to the mandrel deformation, resulting from the temperature change and the incomplete matching of the two FBGs. As expected, using two FBGs could compensate for the temperature, but the output voltage for the sensor configuration with one FBG decreased quickly (red point in Figure 7b).

When the sensor consisted of the mandrel and one FBG, the spectrum shifted due to the temperature change, but the spectrum of the tunable laser was fixed. Therefore, the sensor output changed rapidly.

## 7. Angular Dependence

The acoustic pressure strength decreases with distance [32]. It is preferable that the sensor only varies with one parameter. Thus, eliminating the direction dependence is an advantage.

**Figure 8:**
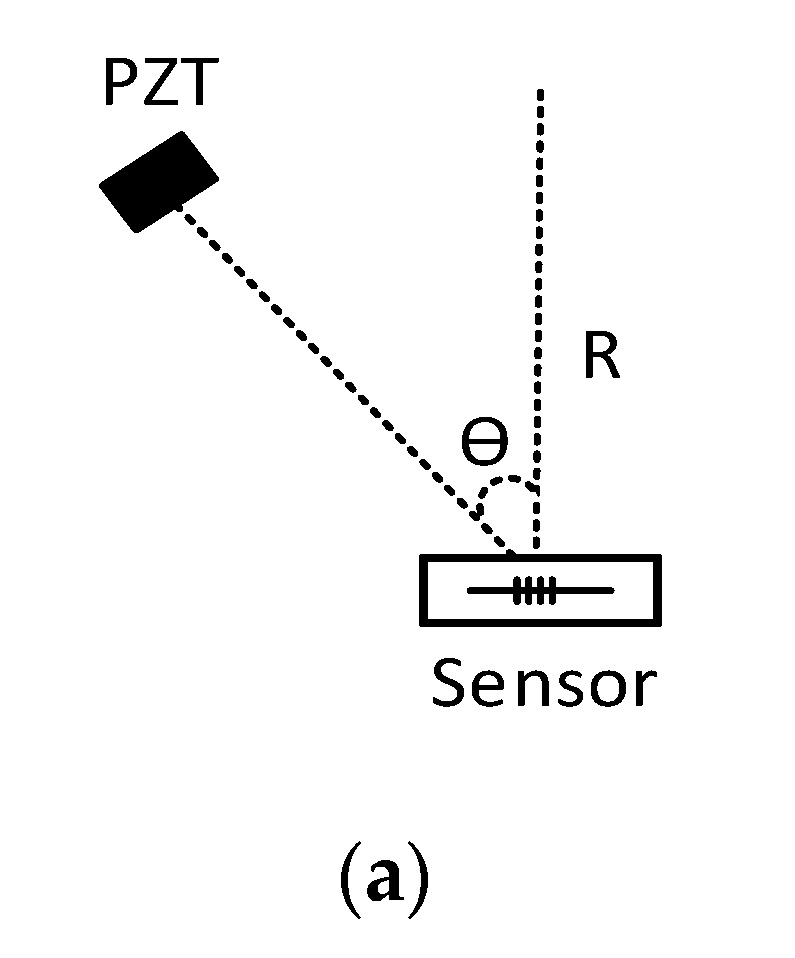

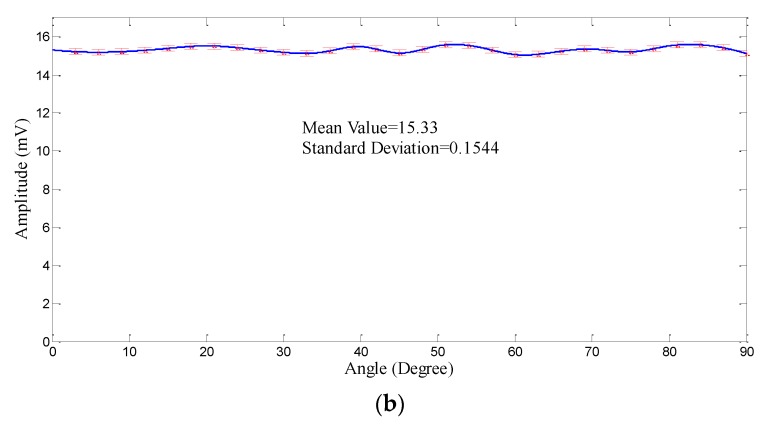
Sensor output due to angular changes. (**a**) The relative position of the piezoelectric transducer and sensor. (**b**) Sensor output voltage at different angles for an acoustic frequency of 193 kHz.

An acoustic wave with a frequency of 193 kHz was generated by the piezoelectric transducer at different theta angles, as depicted in Figure 8a. The source direction varied from zero to 90 degrees in steps of 3 degrees. This test was repeated 10 times, and discrete data converted to the curve of Figure 8b by gathering and interpolating the data. The standard deviation of the output values was approximately 0.4 mV (see Figure 8) [33], which was desirable. The use of an FBG alone completely depends on the direction [34]. In fact, by simultaneously using the FBG and mandrel, this problem could be addressed.

## 8. PD Detection

**Figure 9:**
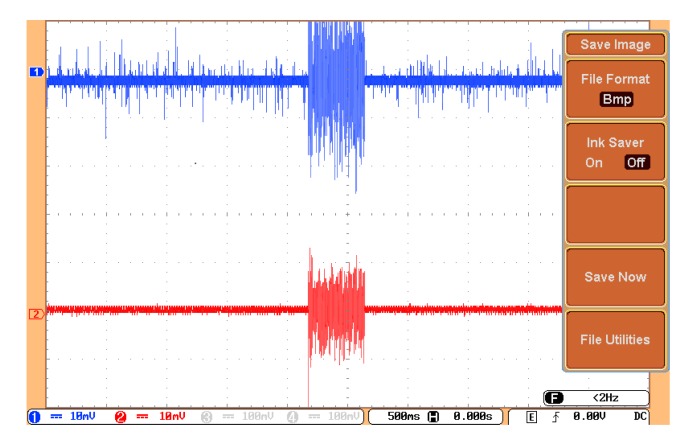
Detection of partial discharge (PD) by the sensor (blue) and piezoelectric transducer (red).

The proposed sensor utilized an amplified acoustic signal and detected the signal over a broad frequency band, as shown in Section 5. To verify the sensor performance, the sensor must be able to identify PD. The piezoelectric transducer was used for the verification of the proposed sensor output. The noise in the proposed sensor was low because there was no electrical measurement system inside the transformer. The transformer was filled with transformer oil, and only the proposed sensor and piezoelectric were placed inside the transformer. The signal was transferred to the electronic system by an optical fiber, which was electromagnetically noiseless. The electronic amplifier, such as an OPAMP, and the oscilloscope, were far from the sensor. An algorithm [34] was also used to reduce the noise and increase the SNR to improve the quality of the signal. The sensor was located 10 cm from the PD source in the oil-filled transformer model. The piezoelectric transducer was positioned beside the sensor, so one expected to detect the same acoustic signal by the sensor and piezoelectric transducer. The signal detected by the sensor and the piezoelectric transducer is depicted in Figure 9. This figure shows that the start and stop times are exactly at the same moment.

**Figure 10:**
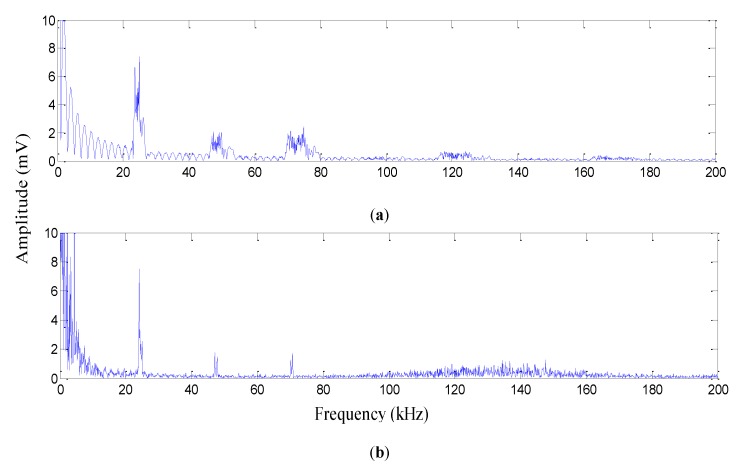
FFT of the output voltage of (**a**) the PD signal from the piezoelectric transducer and (**b**) the PD signal from the proposed FBG sensor.

The frequency of the PD acoustic signal depends on the type, intensity, and distance of the electrodes. At 23 kHz, 45.5 kHz, and 69.6 kHz, we observed three local maxima in the FFT analysis; therefore, these frequencies generated by PD were detected by the sensor (Figure 10b). These frequencies were the second, third, and fourth harmonics of the fundamental frequency of 23 kHz. Likewise, the PD signal detected by the piezoelectric transducer consisted of the second, third, and fourth harmonics of the fundamental frequency of approximately 23 kHz. It is noticeable that the test was conducted on a disconnected transformer. Therefore, there was no electromagnetic noise in the area. The comparison of the results for the piezoelectric transducer and proposed sensor in the time and frequency domains showed the proper performance of the proposed sensor.

## 9. Conclusions

In this study, a new sensor was proposed to detect PD acoustic signals and prevent damage to the transformer. The aim of the proposed sensor was to achieve high-resolution PD detection that is independent of temperature and direction. The ability of the sensor to detect acoustic waves in liquids was studied and characterized in terms of the frequency response, sensitivity, and direction and temperature dependencies. Additionally, experimental tests were carried out to further verify the efficiency of the mandrel and FBG combination. The sensor could detect at least 27 Pa of static pressure and 35 Pa of dynamic pressure using two FBGs. The proposed sensor had a linear response to static pressure and a high resolution to dynamic pressure. The sensor had several resonance frequencies, and the range for favorable detection was around 53 kHz. The temperature dependence of the sensor was less than 0.5 mV for a temperature variation of 14 degrees Celsius. This specification was achieved by employing two FBGs in transmission and reflection modes to compensate temperature variations. The direction dependence was less than 0.4 mV, which was suitable for PD localization. Due to the dependence of the output on distance, it was desirable to utilize a sensor that was independent of direction. In the best result, a PD signal was successfully detected, and the PD frequencies were obtained in the frequency domain, which showed the validity of the results. Future studies on this topic are required to establish the optimal sensor design and placement inside the transformer.
